# Molecular Mechanisms of Same TCM Syndrome for Different Diseases and Different TCM Syndrome for Same Disease in Chronic Hepatitis B and Liver Cirrhosis

**DOI:** 10.1155/2012/120350

**Published:** 2012-05-30

**Authors:** Zhizhong Guo, Shuhao Yu, Yan Guan, Ying-Ya Li, Yi-Yu Lu, Hui Zhang, Shi-Bing Su

**Affiliations:** ^1^Research Center for Complex System of Traditional Chinese Medicine, Shanghai University of Traditional Chinese Medicine, 1200 Cailun Road, Shanghai 201203, China; ^2^College of Life Science and Biotechnology, Shanghai Jiaotong University, 800 Dongchuan Road, Shanghai 200240, China

## Abstract

Traditional Chinese medicine (TCM) treatment is based on the traditional diagnose method to distinguish the TCM syndrome, not the disease. So there is a phenomenon in the relationship between TCM syndrome and disease, called Same TCM Syndrome for Different Diseases and Different TCM Syndrome for Same Disease. In this study, we demonstrated the molecular mechanisms of this phenomenon using the microarray samples of liver-gallbladder dampness-heat syndrome (LGDHS) and liver depression and spleen deficiency syndrome (LDSDS) in the chronic hepatitis B (CHB) and liver cirrhosis (LC). The results showed that the difference between CHB and LC was gene expression level and the difference between LGDHS and LDSDS was gene coexpression in the G-protein-coupled receptor protein-signaling pathway. Therein genes GPER, PTHR1, GPR173, and SSTR1 were coexpressed in LDSDS, but not in LGDHS. Either CHB or LC was divided into the alternative LGDHS and LDSDS by the gene correlation, which reveals the molecular feature of Different TCM Syndrome for Same Disease. The alternatives LGDHS and LDSDS were divided into either CHB or LC by the gene expression level, which reveals the molecular feature of Same TCM Syndrome for Different Diseases.

## 1. Introduction

Traditional Chinese medicine (TCM) is a medical system with at least 3000 years of uninterrupted clinical practice in China. The TCM practice usually requires a TCM syndrome identification based on clinical manifestation followed by the use of individualized treatment that is adapted to address the particular TCM syndrome in patient [1]. Therefore, TCM syndrome, also called ZHENG or TCM pattern, is the core of diagnosis and treatment in TCM [2]. Nowadays, TCM syndrome had been studied in some specific disease such as hypertension [3], coronary heart disease [4], and rheumatoid arthritis [5] or biomedical condition such as neuro-endocrine-immune network [6], suggesting that TCM syndromes are significantly associated with diseases.

Hepatitis B is a viral infection that attacks the liver and can cause both acute and chronic disease. Beyond 25% of hepatitis B virus-infected patients would die of severe chronic liver diseases such as liver cirrhosis and liver cancer [7]. Chronic hepatitis B (CHB) and liver cirrhosis (LC) are the intractable diseases that remain a major public health problem worldwide. Although several antiviral drugs had been approved for CHB, they caused significant side effects and drug resistance. In contrast, TCM treatment was regarded as a safe and effective method for CHB and Liver fibrosis [8, 9].

TCM treatment is based on the traditional diagnose method to differentiate the TCM syndrome, not the disease in western medicine. Therefore, TCM syndromes could be classified in CHB as well as in LC. Moreover, different patients, respectively, suffering CHB or LC could also belong to the same TCM syndrome. This phenomenon is called Same TCM Syndrome for Different Diseases and Different TCM syndrome for Same Disease [10–12]. This viewpoint in TCM is very different with Western medicine. The molecular mechanism of this phenomenon is still a mystery.

Previous study reported liver-gallbladder dampness-heat syndrome (LGDHS) and liver depression and spleen deficiency syndrome (LDSDS) are the major syndromes in CHB [13, 14]. In this study, the aim is to demonstrate the molecular mechanism of Same TCM Syndrome for Different Diseases and Different TCM Syndrome for Same Disease by the analysis of whole gene expression in the same syndrome as LGDHS or LDSDS of different diseases as CHB and LC and the same disease as CHB or LC of different syndromes as LGDHS and LDSDS.

## 2. Material and Methods

### 2.1. Samples

Blood samples from 92 patients were obtained. Therein 14 samples from 2 LGDHS and 3 LDSDS in CHB patients, 3 LGDHS and 3 LDSDS in LC patients and 3 healthy peoples were used to microarray test, and 78 samples from 20 LGDHS and 18 LDSDS in CHB patients, and 21 LGDHS and 19 LDSDS in LC patients were used to test and verify the accuracy of the result. All patients were from Shanghai Longhua Hospital and have signed an agreement with us. The blood samples were morning fasting venous blood and saved in −20°C with 150 *μ*L EDTA.

### 2.2. RNA Extraction and Microarrays

Total RNA of leukocyte from the whole blood was extracted using TRIzol Reagent (Invitrogen, Carlsbad, CA, USA), and a quality control was carried out with NanoDrop ND-1000. The cDNAs were synthesized by the Invitrogen First-Strand cDNA Synthesis kits (Invitrogen, Carlsbad, CA, USA), and RNA polymerase was added to degrade RNA. The cDNA was labeled and hybridized using NimbleGen Homo sapiens 12x135K Arrays (Roche NimbleGen, Madison, WI, USA), according to the manufacturer's protocol.

### 2.3. Real-Time RT-PCR

 Difference-expressed mRNAs were verified by real-time RT-PCR according to SYBR Green Realtime PCR Master Mix kit (TOYOBO, Osaka, Japan) manufacturer. The primer sequences were F: TGGTGTGCGCAGCCATCGTG, R: GCCAGTAACCGGCCACCTCG for DRD5; F: GCTCTGTCAGGGCTCAACCTCC, R: GGCACAAACTTGGAGAGACCGAGC for GABRA; F: GCTACGTGGCCGTGGTGCAT, R: CCGCGGTGCGAGAGAAGACC for SSTR1; F: AGCGAACCCCTCCCACCACA, R: CAGGAAGGCTTGGCTCCGGC for NPFF. F: ACAGAGCCTCGCCTTTGCCG, R: ACATGCCGGAGCCGTTGTCG for ACTB.

### 2.4. Microarray Data Preprocessing and Statistic Analysis

 Microarray data preprocessing was performed using the GenePix software. Raw expression data were log⁡⁡2 transformed and normalized by quantile normalization. Probes were considered robustly expressed if Signal/Noise (SNR) < 2.

We took the average of 3 healthy people in every probe and let every patient sample *ratio* be this average in every probe. In all the following pages: CHB means chronic hepatitis B versus normal; LC means liver cirrhosis versus normal; LGDHS means liver-gallbladder dampness-heat syndrome versus normal; LDSDS means spleen deficiency syndrome versus normal.

The *t*-test function in R software was used to select difference expressed gene (threshold: *P* value < 0.01 or *P* value < 0.05) in diseases between CHB and LC as well as in TCM syndromes between LGDHS and LDSDS. GO enrichment analysis was executed using the selected genes.

 Heatmap analysis, also executed in R, was computing the hierarchical clustering in both rows and columns according to the set of gene values and drawing a color image as a visible result.

The correlation analysis was used to analyze the correlation of difference expressed genes between CHB and LC or LGDHS and LDSDS. The level of significance was set at correlation coefficient >0.5.

### 2.5. Gene Module Analysis and Difference Coexpression Analysis

The Weighted Correlation Network Analysis (WGCNA) R package was used to run the gene module analysis (parameter: networkType = signed, detectCutHeight = 0.97). WGCNA was a systems biology method to describe the correlation patterns among genes across microarray samples. It was used to find clusters (modules) of highly correlated genes and summarizing the clusters using the Module Eigengene (ME) [15].

Furthermore, coXpress R package was used to analyze the difference coexpression (parameter: *s* = pearson, *m* = average, *h* = 0.4). coXpress as a tool has been applied to identify groups of genes that display differential coexpression patterns in microarray datasets and its utility [16].

## 3. Results and Discussion

### 3.1. Difference Expression Analysis

**Figure 1:**
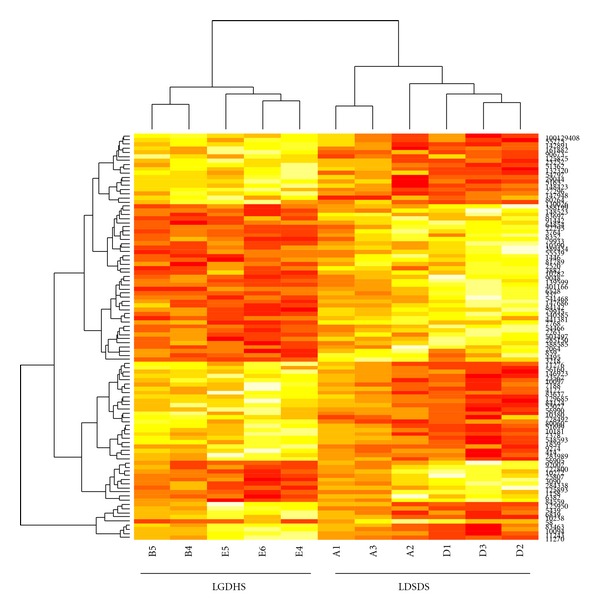
Heatmap of 98 differentially expressed genes between LGDHS and LDSDS. The 98 differentially expressed genes between LGDHS and LDSDS were obviously divided out by Heatmap analysis. Row: genes; column: patient number; deep colour: upexpressed genes; light colour: down-expressed genes; A1–3 and D1–3: LDSDS; B 4, 5 and E4–6: LGDHS.

 At first, to find whether there were some significant genes that could characterize the difference between two disease and two TCM syndromes, *t*-test was used to select difference expression gene in both disease and TCM syndrome levels. The threshold was *P* value less than 0.01. Remarkably, 6579 in all 14352 genes were differentially expressed between CHB and LC, suggested that the difference in mRNA expression level was very clear, according to CHB and LC that were completely different diseases. In contrast, only 98 genes were differentially expressed between LGDHS and LDSDS. The heatmap of the 98 genes between LGDHS and LDSDS was showed in Figure 1. Moreover, though these genes were obviously differentiated into two syndromes, the 98 genes were in disorder, no significantly related function was found by GO enrichment analysis. It also was tried to change the threshold as *P* value less than 0.05 and got 830 genes, but still any significantly related GO function was not found.

### 3.2. Gene Modules Related with Disease or TCM Syndrome

**Figure 2:**
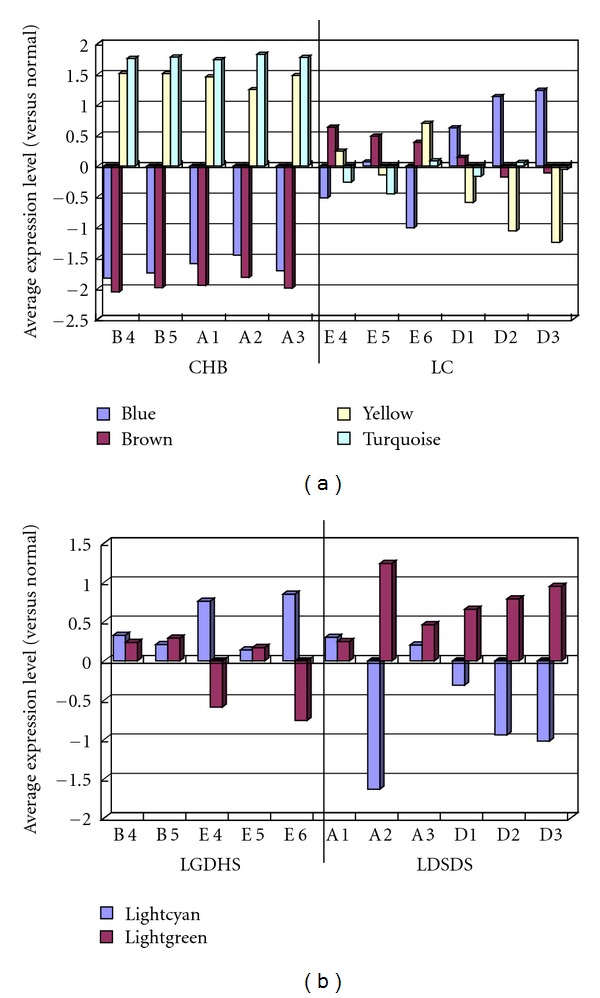
Average gene expression in modules which correlated with diseases or TCM syndromes. In the diseases (a), blue and brown modules both had low expression value in CHB and not consistent in LC. Yellow and turquoise modules both had high expression value in CHB and not consistent in LC. In the TCM syndromes (b), lightcyan modules had low expression value in LDSDS. Lightgreen modules had high expression value in LDSDS. A1–3 and D1–3: LDSDS; B 4, 5 and E4–6: LGDHS.

 Due to the above result that the molecular mechanisms of the difference between two TCM syndromes could be not commendably explained with the single-gene difference expression method, then the gene module method was used to demonstrate the difference between diseases and TCM syndromes. The all 14352 genes were taken into 26 gene modules by the WGCNA R package [15], and each module had a name of color and a ME to identify the gene expression. Among the 26 modules, some significant modules were screened out by correlating the MEs in our disease trail or TCM syndrome trail. In the result, blue, brown, turquoise, and yellow modules were most related with the difference between CHB and LC (Figure 2(a)), and lightgreen module and lightcyan module were most related with the difference between LGDHS and LDSDS (Figure 2(b)).

The above 6 gene modules were used to GO enrichment analysis. The result showed that the blue module was mainly enriched in G-protein-coupled receptor protein-signaling pathway, brown module was mainly enriched in immune system process, yellow module was mainly enriched in cell cycle, and turquoise module was enriched in many basal metabolisms. But it was still hard to understand that ossification function was enriched in lightcyan module, and the lightgreen module did not enrich in any GO function module.

### 3.3. Comparing Difference Coexpression Network between Two TCM Syndromes

**Figure 3:**
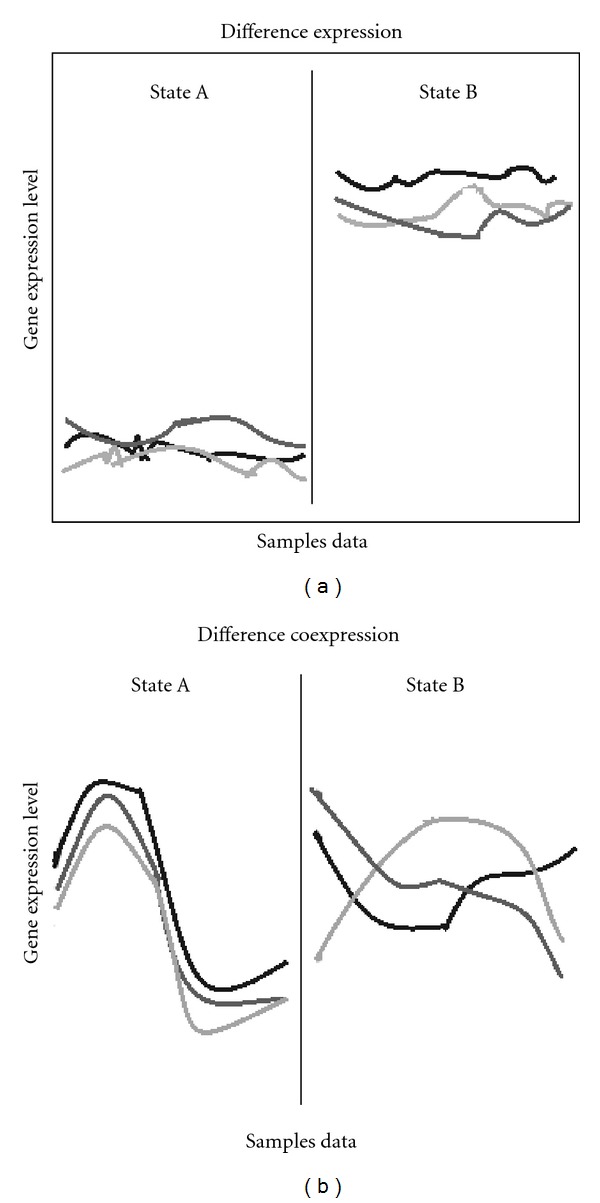
Schematic diagram of difference expression and difference coexpression. Graph of the difference expression (a) represented that there are genes different expression levels between states A and B, and the difference coexpression (b) represented that there is higher correlation in state A and lower correlation in state B. Curves were represented as whichever genes.

 To further demonstrate the mechanism of difference between two TCM syndromes, the correlation of gene expression including difference expression and difference coexpression was analyzed.  Figure 3 was a schematic diagram which showed the meaning of difference expression or difference coexpression, respectively. The difference expression meant that there were gene different expression levels between two states. The difference coexpression meant that there was higher gene correlation in a state and lower gene correlation in another state.

Then, the difference coexpression groups between LGDHS and LDSDS were analyzed using the advantage of coXpress R package [16]. First, through the analysis using the 830 differential expression genes (*P* < 0.05 in *t*-test) between the LGDHS and LDSDS, the gene groups whose gene members were coexpressed in LGDHS and not co-expressed in LDSDS were produced by coXpress (A in Table 1). Then we also executed the coXpress again to find the gene groups whose gene members were coexpressed in LDSDS and not coexpressed in LGDHS (B in Table 1). The *P* values including p.g1 in and p.g2 indicated a gene confusion degree in every group in LGDHS or LDSDS, respectively, (*P* > 0.05 was jumbled or not coexpressed; *P* < 0.05 was order or coexpressed).

**Figure 4:**
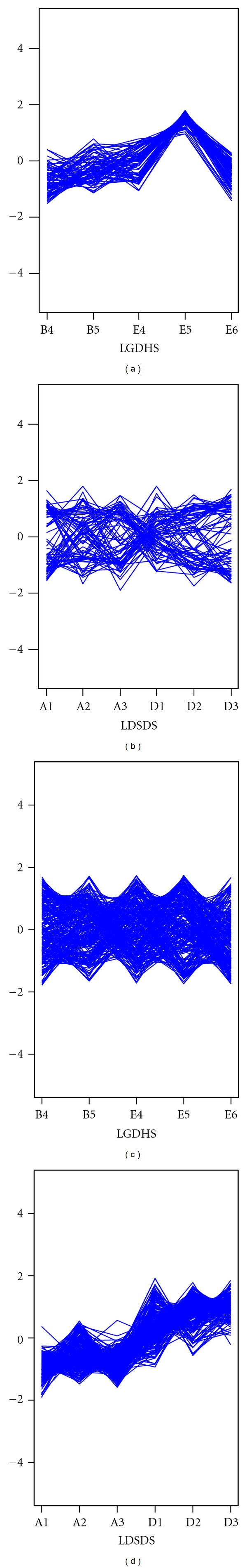
The gene confusion degree of group 2 and 9 in LGDHS and LDSDS. CoXpress was used to find orderly gene groups in LGDHS or LDSDS. The genes in group 9 of orderly gene groups in LGDHS showed good consistency in LGDHS (a) and poor consistency in LDSDS (b). The genes in group 2 of orderly gene groups in LDSDS showed poor consistency in LGDHS (c) and good consistency in LDSDS (d). A1–3 and D1–3: LDSDS; B 4, 5 and E4–6: LGDHS.

It was found that the gene coexpression groups were orderly in LGDHS but jumbled in LDSDS (A in Table 1). Among the groups jumbled in LDSDS, There were the most gene numbers in group 9. The gene confusion degree in group 9 was showed in Figure 4. It was observed that genes of LGDHS in group 9 had similar traces (Figure 4(a)), whereas the traces of LDSDS were varied (Figure 4(b)). To further clarify the functional mechanism at molecular level, GO enrichment analysis was taken on the genes in group 9. As Table 2 revealed, LGDHS was involved in electron transport chain function, but LDSDS does not.

Analogously, it was also found that the gene coexpression groups were orderly in LDSDS but jumbled in LGDHS (B in Table 1). Among the groups jumbled in LGDHS, there were the most gene numbers in group 2. Therefore, group 2 were analyzed and showed that the traces of LGDHS were varied (Figure 4(c)) and the traces of LDSDS were in order (Figure 4(d)). Through further studied the molecular functional mechanism by the GO enrichment analysis, it was found that LDSDS was involved in G-protein-coupled receptor protein-signaling pathway (GCRP pathway), but LGDHS does not (Table 2).

### 3.4. Molecular Mechanism of Difference between Diseases and TCM Syndromes

**Figure 5:**
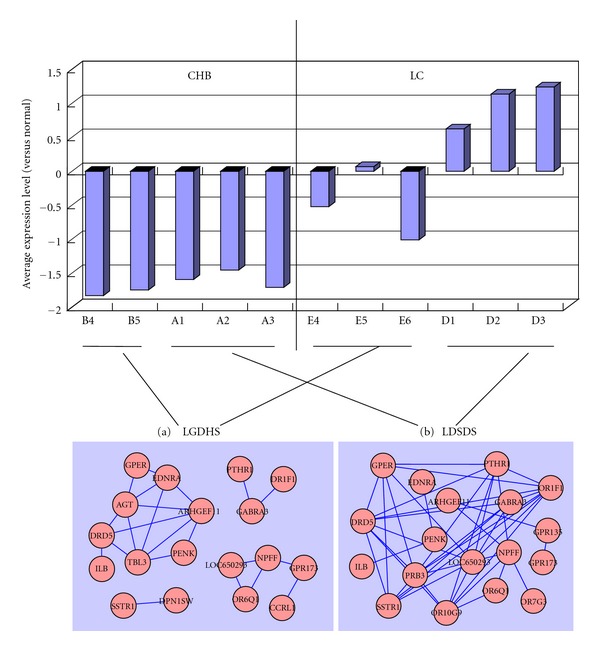
Gene relationships in GCRP pathway in diseases and TCM syndromes. GO enrichment analysis of genes in group 2 was carried out. Whether diseases (CHB or LC) and TCM syndromes (LGDHS or LDSDS) were correlated to GCRP pathway, the gene expression (upper figure) was represented that the gene expression levels were lower in CHB and higher or lower in LC. The gene network ((a), (b)) was represented that the genes connections in LDSDS (b) were more than LGDHS (a).

 It was interesting in our result that the genes coexpression in group 2 was enriched in GCRP pathway. Because same situation happened to the genes in blue module, which was related with the difference between CHB and LC by the gene module analysis, these genes in GCRP pathway were differentially expressed between CHB and LC and difference coexpressed between LGDHS and LDSDS. These results were summarized in Figure 5. Interestingly, in GCRP pathway, whether TCM syndrome was LGDHS or LDSDS, the gene expression level was lower in CHB and higher or lower in LC, and whether disease was CHB or LC, the genes in LDSDS had higher correlation than LGDHS. For example, in LDSDS, genes GPER, PTHR1, GPR173, and SSTR1 were connected in a correlation network together, while they, respectively, belong to four correlation networks in LGDHS (Figure 5). These results suggested the different molecular mechanism between diseases (CHB and LC) and TCM syndromes (LGDHS and LDSDS).

### 3.5. Average Expression and Correlation of DRD5 GABRA SSTR1 and NPFF Genes in Diseases and TCM Syndromes

**Figure 6:**
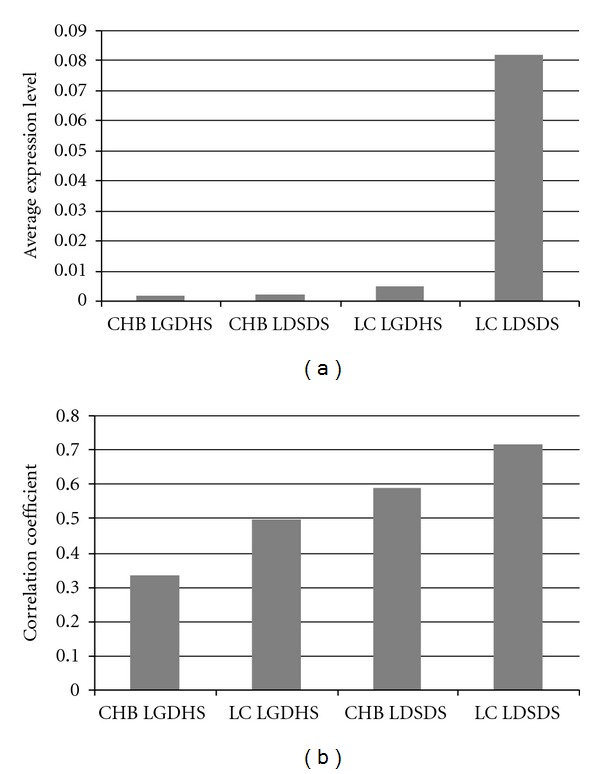
Average expression and correlation of DRD5 GABRA SSTR1 and NPFF mRNAs in diseases and TCM syndromes. The gene expression levels of both LGDHS and LDSDS were lower in CHB and that of LDSDS was more than LGDHS in LC (a). (Gene expression levels were the ratio of each mRNA and ACTB mRNA). The correlation coefficient of LDSDS in CHB and LC was more than LGDHS in CHB and LC (b).

 To test and verify the difference of average expression level and correlation of genes in GCRP pathway, DRD5 GABRA SSTR1 and NPFF mRNAs were expressed by real-time RT-PCR. The average expression levels of these genes in both LGDHS and LDSDS were lower in CHB, and that of LDSDS was more than LGDHS in LC (Figure 6(a)). The correlation coefficient of LDSDS (>0.5) in CHB and LC was more than LGDHS (<0.5) in CHB and LC (Figure 6(b)). These results further confirmed that the gene expression level was lower in CHB and higher or lower in LC. The genes in LDSDS had higher correlation than LGDHS whether disease was CHB or LC.

Previous researches had also found that LC was related with GCRP pathway [17–19], but little literature touched upon the relation between CHB and GCRP. Our result also indicated that genes in GCRP pathway were higher expression in LC and lower expression in CHB. It suggested that LC was a more serious disease than CHB by the activity of GCRP pathway. Further research will clarify the role of genes in GCRP pathway from CHB develop to LC.

Interestingly, our results showed that TCM syndromes, LGDHS and LDSDS did not clearly relate with the gene expression levels in GCRP pathway. The genes correlation or cooperation was more important. As shown in Figure 4, the genes in LDSDS had more connections than LGDHS, so LGDHS and LDSDS constructed different gene network. It incarnated the holistic thought in TCM.

Therefore, our research results suggested that CHB could be divided into LGDHS and LDSDS by the gene correlation as well as LC, which reveals the molecular feature of Different TCM Syndrome for Same Disease. Analogously, LGDHS was being in CHB or LC by the gene expression level as well as LDSDS, which reveals the molecular feature of Same TCM Syndrome for Different Diseases. The schematic diagram of the molecular mechanisms was showed in Figure 2.

There are two kinds of therapeutic principles in the TCM syndrome identification and treatment process, called Different treatments for the same disease and same treatment for different diseases. The Different treatments for the same disease means using different prescriptions or Chinese herbal medicines to treat the different TCM syndromes in the same disease process. The Same treatment for different diseases means using the same and prescriptions or Chinese herbal medicines to treat the same TCM syndrome in different disease process. These therapeutic principles are widely used in TCM practice as personalized therapy [12, 20]. Therefore, understanding the molecular mechanisms of Same TCM Syndrome for Different Diseases and Different TCM Syndrome for Same Disease will be primely serving for TCM diagnosis and treatment. This research provided firstly the evidence. Further research will be required more samples to proving this evidence.

## 4. Conclusion

The classification of TCM syndrome is a diagnostic method. TCM syndromes are significantly associated with diseases, which are involved in Same TCM Syndrome for Different Diseases and Different TCM Syndrome for Same Disease. In this study, through analyzing microarray date of LGDHS and LDSDS in patients with CHB and LC, we provided evidence that the difference between CHB and LC was gene expression and the difference between LGDHS and LDSDS was gene coexpression in G-protein-coupled receptor protein-signaling pathway. Therein genes GPER, PTHR1, GPR173, and SSTR1 were coexpressed in LDSDS but not in LGDHS. Either CHB or LC was divided into the alternative LGDHS and LDSDS by the gene correlation, which reveals the molecular feature of Different TCM Syndrome for Same Disease. Either LGDHS or LDSDS was divided into the alternative CHB and LC by the gene expression level, which reveals the molecular feature of Same TCM Syndrome for Different Diseases. These results might be significant for both TCM research and TCM diagnosis and treatment.

## Figures and Tables

**Table 1:** Comparison of gene coexpression groups in LGDHS and LDSDS.

Group ID	Gene number	P.g1	P.g2
	A LGDHS		
8	6	0.00	0.62
5	10	0.00	0.31
***9***	***81***	***0.00***	***0.83***
14	18	0.00	0.38
12	34	0.00	0.11
17	15	0.00	0.05
13	45	0.00	0.14
10	58	0.00	0.03
4	19	0.00	0.15
16	27	0.00	0.02
15	55	0.00	0.00
3	48	0.00	0.00
6	16	0.00	0.01
11	92	0.00	0.00
2	11	0.00	0.00
1	234	0.00	0.00
7	61	0.00	0.00

	B LDSDS		

9	6	0.00	0.00
17	10	0.00	0.00
12	13	0.01	0.00
7	297	0.00	0.00
14	5	0.12	0.00
4	90	0.00	0.00
8	5	0.20	0.00
10	12	0.04	0.00
5	69	0.53	0.00
6	26	0.83	0.00
15	3	0.49	0.08
***2***	***238***	***0.69***	***0.00***
3	21	0.87	0.00
11	8	0.54	0.00
1	8	0.36	0.00
13	4	0.62	0.05
18	6	0.83	0.00
16	9	0.76	0.07

**Table 2:** GO enrichments of orderly group 2 in LDSDS and group 9 in LGDHS.

GO term ID	Orderly group	Enrichment *P*	Term name
GO:0006120	LGDHS 9	0.022478	Mitochondrial electron transport, NADH to ubiquinone
GO:0022900	LGDHS 9	0.022478	Electron transport chain
GO:0022904	LGDHS 9	0.022478	Respiratory electron transport Chain
GO:0042773	LGDHS 9	0.022478	ATP synthesis coupled electron transport
GO:0042775	LGDHS 9	0.022478	Organelle ATP synthesis coupled electron transport
GO:0006119	LGDHS 9	0.04236	Oxidative phosphorylation
GO:0010468	LGDHS 9	0.048855	Regulation of gene expression
GO:0009987	LGDHS 9	0.049535	Cellular process
GO:0016070	LGDHS 9	0.059695	RNA metabolic process
GO:0006355	LGDHS 9	0.061016	Regulation of transcription, DNA-dependent

GO:0007186	LDSDS2	0.000668	G-protein coupled receptor protein signaling pathway
GO:0007606	LDSDS2	0.004518	Sensory perception of chemical stimulus
GO:0007608	LDSDS2	0.004518	Sensory perception of smell
GO:0007166	LDSDS2	0.014079	Cell surface receptor linked signal transduction
GO:0007586	LDSDS2	0.015106	digestion
GO:0007223	LDSDS2	0.017534	Wnt receptor signaling pathway, calcium modulating pathway
GO:0008203	LDSDS2	0.017534	Cholesterol metabolic process
GO:0016125	LDSDS2	0.017534	Sterol metabolic process
GO:0042157	LDSDS2	0.017534	Lipoprotein metabolic process
GO:0006813	LDSDS2	0.017952	Potassium ion transport
